# Hypertension in Patients Treated With Ibrutinib for Chronic Lymphocytic Leukemia

**DOI:** 10.1001/jamanetworkopen.2019.16326

**Published:** 2019-12-02

**Authors:** Lindsey E. Roeker, Maryam Sarraf Yazdy, Joanna Rhodes, Julie Goodfriend, Mayur Narkhede, Joseph Carver, Anthony Mato

**Affiliations:** 1 Department of Medicine, Memorial Sloan Kettering Cancer Center, New York, New York; 2 Division of Hematology and Oncology, Department of Medicine, Georgetown University Hospital Lombardi Comprehensive Cancer Center, Washington, DC; 3 Department of Hematology and Oncology, Northwell Health Cancer Institute, New Hyde Park, New York; 4 Division of Hematology and Oncology, Department of Medicine, University of Alabama, Birmingham; 5 Cardiovascular Division, Department of Medicine, Abramson Cancer Center, University of Pennsylvania, Philadelphia; 6 Division of Hematologic Malignancies, Department of Medicine, Memorial Sloan Kettering Cancer Center, New York, New York

## Abstract

This cohort study describes patterns of development, management strategies, and long-term vascular consequences of hypertension associated with ibrutinib in the non–clinical trial setting.

## Introduction

Hypertension is a commonly noted adverse event for patients with chronic lymphocytic leukemia who are treated with ibrutinib in the clinical trial setting (incidence, 18%; grade ≥3 hypertension, 6%).^1^ In clinical practice settings, patterns of development of and management strategies for hypertension are less well understood. Thus, we aimed to describe patterns of development, management strategies, and long-term vascular consequences of hypertension associated with ibrutinib in the non–clinical trial setting.

## Methods

This multicenter cohort study took place at the Memorial Sloan Cancer Center in New York, New York; the Lombardi Cancer Center, Georgetown University Hospital, in Washington, DC; and the Abramson Cancer Center, University of Pennsylvania, in Philadelphia. It included patients treated with 420 mg of ibrutinib daily for at least 6 months outside of clinical trials; patients requiring dose reductions were excluded. Baseline cardiovascular comorbidities and medication regimens, sequential blood pressure (BP) measurements before and after ibrutinib exposure, adjustments to cardiovascular medication regimens, and development of cardiovascular complications were collected. Institutional review boards at all institutions approved the study, and informed consent was not required because of minimal risk to participants. This study followed the Strengthening the Reporting of Observational Studies in Epidemiology (STROBE) reporting guideline.

The *t* test was used to compare medians. Univariate analysis was performed to assess associations of baseline features with the development of hypertension. Other statistics were descriptive. Statistical significance was set at *P* < .05, and all tests were 2-tailed. Statistical analysis was performed using Stata statistical software version 10.1 (StataCorp).

## Results

We identified 247 patients at 3 institutions treated with ibrutinib. Before initiation of ibrutinib, baseline cardiovascular comorbidities included hypertension (107 [43.3%]), hyperlipidemia (87 [35.2%]), diabetes (40 [16.2%]), coronary artery disease (24 [9.7%]), heart failure (5 [2.0%]), valvular heart disease (7 [2.8%]), atrial fibrillation (11 [4.5%]), other arrhythmia (16 [6.5%]), stroke (7 [2.8%]), and smoking history (104 [42.1%]). Details regarding baseline cardiovascular medication profiles are available in the Table. Median (range) systolic BP before ibrutinib exposure was 127 (90-182) mm Hg, and median (range) diastolic BP was 71 (48-95) mm Hg, with 72 of 107 patients (67.3%) who were diagnosed with hypertension having well-controlled baseline BP (ie, <140/90 mm Hg).

**Table.** Cardiovascular Medication Regimens at Baseline and Within the First Year of Ibrutinib Exposure Among 247 Patients With Chronic Lymphocytic Leukemia^a^

Medication	Patients, No./Total No. (%)	
	Baseline	After Ibrutinib Exposure
Cardiovascular agents		
Median (range), No.	1 (0-7)	1 (0-8)
Any	125/245 (51.0)	140/246 (56.9)
≥2	82/245 (33.5)	87/245 (35.5)
β-blocker	49/246 (19.9)	65/207 (31.4)
Calcium channel blocker	29/245 (11.8)	30/199 (15.1)
ACE inhibitor or ARB	73/246 (29.7)	84/211 (39.8)
Diuretic	30/246 (12.2)	36/204 (17.6)
Aspirin	54/246 (22.0)	50/198 (25.3)
Anticoagulation	12/246 (4.9)	18/201 (9.0)
Lipid lowering agent	72/243 (29.6)	66/201 (32.8)
Antiarrhythmic	2/246 (0.8)	7/196 (3.6)

Abbreviations: ACE, angiotensin-converting enzyme; ARB, angiotensin receptor blocker.

^a^ Ibrutinib exposure was 420 mg daily.

The Figure depicts median systolic and diastolic BP measurements in the first year after ibrutinib initiation. Median (range) peak BP following ibrutinib exposure for the entire cohort was significantly elevated from baseline (systolic: 153 [105 to 215] mm Hg; diastolic, 80 [53 to 121] mm Hg; *P* < .001), with a median (range) time to peak BP of 6 (0 to 35) months. Systolic BP changed more than diastolic BP (systolic BP: median [range] change, 21 [−21 to 86] mm Hg; 19% median excursion from baseline; diastolic BP: median [range] change, 8.5 [−14 to 40] mm Hg; 11% excursion from baseline). Incident hypertension (ie, BP ≥140/90 mm Hg) was observed in 86 participants (34.8%), with grade 3 or higher systolic hypertension observed in 82 (33.2%). Of those without baseline hypertension, new systolic hypertension developed in 92 participants (65.7%), and new diastolic hypertension developed in 25 (17.9%). For those with baseline hypertension, 53 (49.5%) experienced systolic hypertension of grade 3 or higher according to the Common Terminology Criteria for Adverse Events, and 5 (4.7%) experienced diastolic hypertension of grade 3 or higher according to the Common Terminology Criteria for Adverse Events. New atrial fibrillation was observed in 16 participants (6.4%), and coronary artery disease was diagnosed in 4 participants (1.6%). Preexisting cardiovascular comorbidities, smoking history, and diabetes were not associated with the development of hypertension in a univariate analysis.

**Figure.**
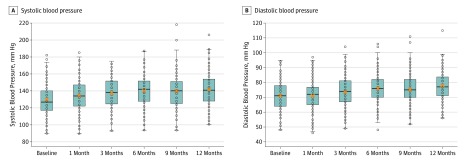
Blood Pressure Before and After Ibrutinib Each patient’s blood pressure reading is depicted by a dot. The box represents the 25th to 75th percentile, with the median noted by a line in the middle of the box and the mean by an orange x. The whiskers represent minimum and maximum values, excluding outliers.

A total of 51 patients (20.6%) had a change in their cardiovascular medication regimen in the first year after ibrutinib exposure. Of these, 15 (29.4%) increased the dose of an existing hypertensive medication, and 41 (80.4%) started at least 1 new antihypertensive medication. Of the 41 patients who started a new medication, 8 (19.5%) started at least 2 new agents. The most common new classes of agents selected were β-blockers (18 participants [43.9%]), diuretics (9 participants [30.0%]), angiotensin-converting enzyme inhibitors (8 participants [19.5%]), angiotensin receptor blockers (8 participants [19.5%]), and calcium channel blockers (7 participants [17.1%]).

## Discussion

Ibrutinib-associated hypertension is a common adverse event affecting those with and without preexisting hypertension.^1,2,3,4^ We found that median time to peak BP was 6 months, suggesting the need for ongoing and close monitoring of BP in patients treated with ibrutinib for several months after drug initiation. The observed rate of incident hypertension and grade 3 or higher systolic hypertension was higher than that observed in clinical trials,^3^ perhaps reflecting the inclusion of a more comorbid population with potentially less stringent monitoring or different management strategies used in practice vs clinical trial settings. The discrepancy between the proportion of patients experiencing new or worsening hypertension and those with a change in their antihypertensive regimens suggested a potential opportunity for care optimization as well as patient and clinician education. This study is limited by its retrospective design. This study, combined with available clinical trial data on hypertension incidence, may be influenced by natural variation in BP, emphasizing the importance of measuring the variability of BP before and after ibrutinib exposure. Because ibrutinib is given continuously and hypertension prevalence is cumulative, ongoing study of long-term vascular consequences of ibrutinib-associated hypertension is needed.
